# Characterization of the Durum Wheat-*Aegilops tauschii* 4D(4B) Disomic Substitution Line YL-443 With Superior Characteristics of High Yielding and Stripe Rust Resistance

**DOI:** 10.3389/fpls.2021.745290

**Published:** 2021-09-30

**Authors:** Fan Yang, Qier Liu, Qin Wang, Ning Yang, Jun Li, Honshen Wan, Zehou Liu, Sujie Yang, Ying Wang, Jie Zhang, Hang Liu, Xing Fan, Wujun Ma, Wuyun Yang, Yonghong Zhou

**Affiliations:** ^1^Triticeae Research Institute, Sichuan Agricultural University, Chengdu, China; ^2^Crop Research Institute, Sichuan Academy of Agricultural Sciences (SAAS), Chengdu, China; ^3^Australia-China Joint Centre for Wheat Improvement, Western Australian State Agricultural Biotechnology Centre, College of Science, Health, Engineering and Education, Murdoch University, Perth, WA, Australia; ^4^Institute of Germplasm Resources and Biotechnology, Jiangsu Academy of Agricultural Sciences (JAAS), Nanjing, China; ^5^Key Laboratory of Wheat Biology and Genetic Improvement on Southwestern China, Ministry of Agriculture, Chengdu, China; ^6^Institute of Biotechnology and Nuclear Technology Research, Sichuan Academy of Agricultural Sciences (SAAS), Chengdu, China

**Keywords:** durum wheat, *Aegilops tauschii*, substitution, pentaploidy, supernumerary spikelets, stripe rust, interspecific hybridization

## Abstract

Durum wheat is one of the important food and cash crops. The main goals in current breeding programs are improving its low yield potential, kernel characteristics, and lack of resistance or tolerance to some biotic and abiotic stresses. In this study, a nascent synthesized hexaploid wheat Lanmai/AT23 is used as the female parent in crosses with its AB genome donor Lanmai. A tetraploid line YL-443 with supernumerary spikelets and high resistance to stripe rust was selected out from the pentaploid F_7_ progeny. Somatic analysis using multicolor fluorescence *in situ* hybridization (mc-FISH) revealed that this line is a disomic substitution line with the 4B chromosome pair of Lanmai replaced by the 4D chromosome pair of *Aegilops tauschii* AT23. Comparing with Lanmai, YL-443 shows an increase in the number of spikelets and florets per spike by 36.3 and 75.9%, respectively. The stripe rust resistance gene *Yr28* carried on the 4D chromosome was fully expressed in the tetraploid background. The present 4D(4B) disomic substitution line YL-443 was distinguished from the previously reported 4D(4B) lines with the 4D chromosomes from Chinese Spring (CS). Our study demonstrated that YL-443 can be used as elite germplasm for durum wheat breeding targeting high yield potential and stripe rust resistance. The *Yr28*-specific PCR marker and the 4D chromosome-specific KASP markers together with its unique features of pubescent leaf sheath and auricles can be utilized for assisting selection in breeding.

## Introduction

Tetraploid durum wheat (2*n* = 28, AABB, *Triticum turgidum* L. ssp. *durum*) appears to have been derived from wild emmer wheat (*T. turgidum* ssp. *dicoccoides*) about 10,000 years ago in southeast Turkey, most likely through artificial domestication and continuous evolution (Özkan et al., 2011; Tidiane Sall et al., 2019). Tetraploid durum subsequently hybridized with diploid *Aegilops tauschii* Coss. (2*n* = 14, DD), resulting in the allohexaploid wheat (*Triticum aestivum* L.) (Kihara, 1944; McFadden and Sears, 1946). The addition of the D genome of *Ae. tauschii* into durum wheat improves the plasticity of hexaploid wheat, conferring a higher adaption capacity than durum wheat, including broader adaptability to different photoperiod and vernalization conditions, improved abiotic tolerance, and increased biotic resistance (Dubcovsky and Dvorak, 2007; Li et al., 2014, 2018). The allopolyploidy also gives hexaploid wheat greater potential to produce a variety of food products, such as bread, biscuits, cakes, pasta, noodles, and even brewing wine (Shewry, 2009; Faltermaier et al., 2014). Compared with bread wheat, durum wheat only accounts for 5% of the cultivated area and is principally grown in the Mediterranean region, Canada, United States, Mexico, India, Australia, etc. (Tidiane Sall et al., 2019; Xynias et al., 2020). Despite its lower planting area compared with hexaploid wheat, the global durum wheat production has been forecast up to 42.7 million tons (MT) in 2020–2021 (Agriculture Agri-Food Canada, 2021). About 75% of the global durum wheat production is contributed by the Mediterranean region (Othmeni et al., 2019), where crops are mainly grown under environmental (high temperature, drought, and salinity) and biotic (pests and diseases) stresses (Tidiane Sall et al., 2019). In China, durum wheat cultivation was first started in the 1970s using varieties introduced from European countries such as Italy (Zhao et al., 2002). It was only grown in China sporadically in the beginning. Since then, the pasta flavor is getting more and more popular particularly among young people, resulting in huge demand for durum wheat. A total of 1.71 and 1.97 MT of durum wheat were imported in 2015 and 2019, respectively (Zhiyan, 2020). The high level of consumption resulted in expanded domestic cultivation. As a consequence, domestic cultivars with high yield and quality are required in the farming sector, making durum wheat breeding an important task in China.

Durum wheat improvement mainly focuses on aspects of kernel hardness, protein content, dough elasticity and extensibility, gluten strength, and yellow pigment concentration (Magallanes-López et al., 2017), which play a key role in processing and end-use quality. Hybrids between hexaploid and durum wheat are readily produced (Padmanaban et al., 2017), and hence, researchers have proposed using hexaploid wheat as a genetic resource to improve durum wheat (Ma et al., 2013; Padmanaban et al., 2017; Mastrangelo and Cattivelli, 2021). To date, a substantial number of hexaploid wheat genes have been successfully introgressed into durum wheat through pentaploids produced by hybridization. The *Glu-D1* locus encoding high molecular weight (HMW) glutenin (Morris et al., 2019; Sissons et al., 2019) was transferred from common wheat mediated by the Chinese Spring (CS) mutant line *ph1b* (Sears, 1977) and durum wheat mutant line *ph1c* (Giorgi, 1978) into 1A chromosome of the recipient durum wheat for improving baking quality (Ceoloni et al., 1995, 1996; Vitellozzi et al., 1997; Gennaro et al., 2012). The endosperm softness control gene Puroindoline (*Pin-a* and *Pin-b*) on 5DS was transferred by *ph1b*-mediated translocation and generated soft kernel durum wheat (Bhave and Morris, 2008; Boehm et al., 2017). The durum wheat cultivar Langdon (LDN) and hexaploid wheat landrace CS have been used to develop a set of D-chromosome disomic substitution lines for transferring desirable genes (Joppa and Williams, 1988). Morris et al. (2011) developed a soft durum wheat by using the LDN 5D(5B) substitution line. The introgression of a 4D-chromosome fragment from the LDN 4D(4B) substitution line increased the Al^3+^ tolerance of durum wheat (Han et al., 2014, 2016). Calderón et al. (2012) successfully developed durum wheat-*Hordeum chilense* introgression lines by crossing a CS 4H^ch^(4B) substitution line with a LDN 4D(4B) substitution line, which may contain *Septoria tritici* blotch resistance and salt tolerance gene. In order to transfer abiotic stress and disease resistance genes from *Amblyopyrum muticum* into durum wheat, Othmeni et al. (2019) utilized bread wheat/*Am. muticum* hybrids to cross with durum wheat, resulting in D-genome and/or T-genome segment introgression lines.

**Figure 1:**
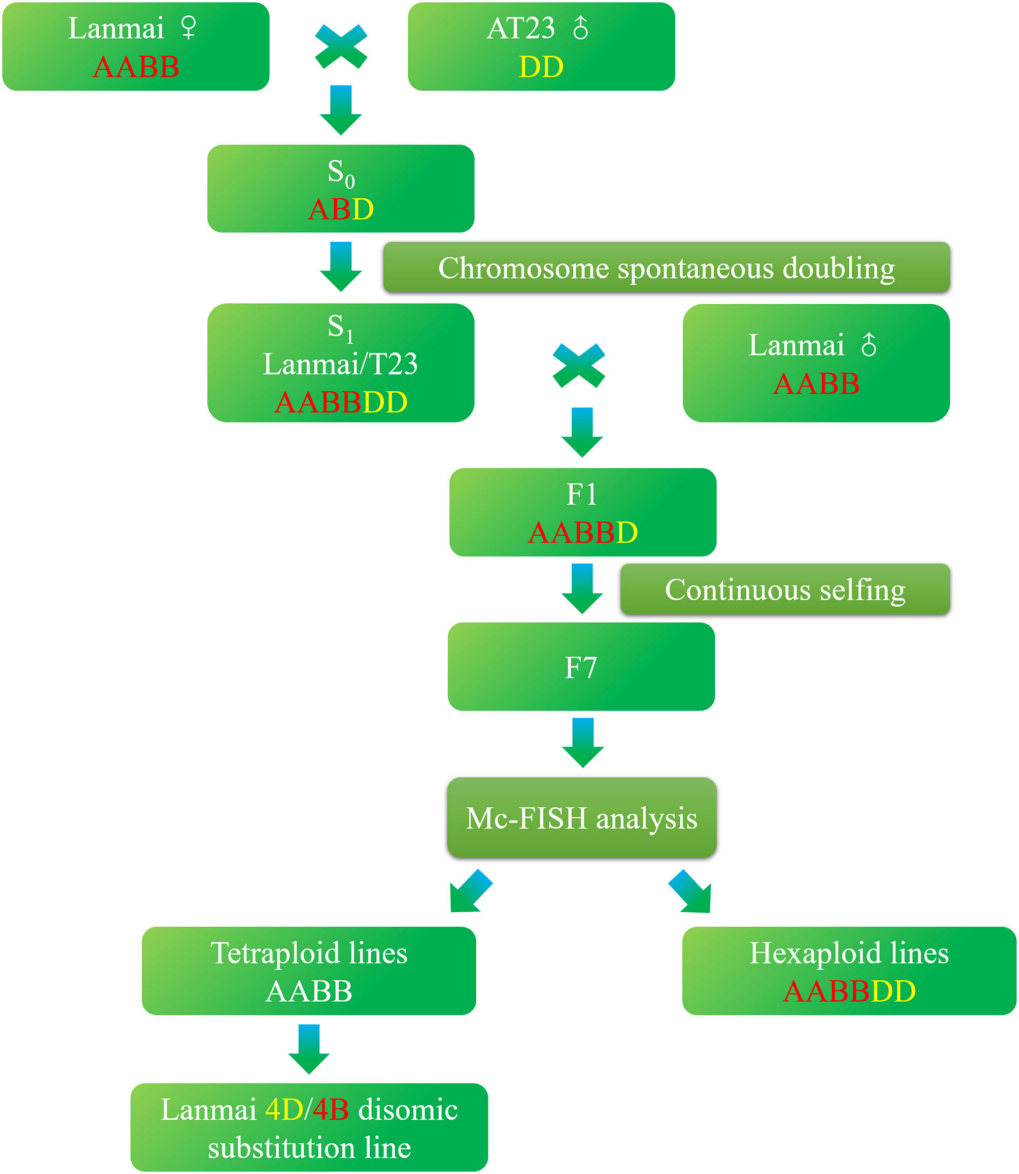
Procedure followed to develop durum wheat Lanmai 4D(4B) disomic substitution line YL-443.

In this research, we first developed a nascent synthetic hexaploid wheat (SHW) Lanmai/AT23 line YL-724 using durum wheat Lanmai and *Ae. tauschii* accession AT23, which was subsequently crossed with Lanmai to produce a 4D(4B) disomic substitution line YL-443 after six consecutive generations of selfing (Figure 1). The developed line displayed supernumerary spikelets and high resistance to stripe rust. It can be used for durum wheat breeding to increase yield potential and disease resistance.

## Materials and Methods

### Production of Hybrids

The development of durum wheat/*Ae. tauschii* introgression lines began by crossing Lanmai (2*n* = 28, AABB) and *Ae. tauschii* accession AT23 (2*n* = 14, DD), followed by spontaneous chromosome doubling. Lanmai is a selected line from LDN (*T. turgidum* L. var. *durum* cv. Langdon) as 3–5 days early flowering. This variation may have been attributed to the spontaneous mutation of LDN or out-crossing of LDN with an unknown cultivar. The euploid SHW lines of Lanmai/AT23 with 42 chromosomes (AABBDD) were identified using multicolor fluorescence *in situ* hybridization (mc-FISH) assay. One of the euploid SHW lines, YL-724, was selected and used as female parent to cross with Lanmai. After crossing, six consecutive cycles of selfing were carried out until the F_7_ generation. Since Lanmai can promote the spontaneous doubling of chromosome of haplotype hybrids (ABD or ABD^v^N^v^) produced by crossing Lanmai with *Ae. tauschii* or *Aegilops ventricosa* (Hao et al., 2014; Zhang et al., 2020), the production of F_1_ hybrids did not require embryo rescue techniques or hormone treatment. During selfing, spikes of F_2_ hybrid plants were randomly selected and planted in rows. Individual plants of the F_3_ generation with desirable yield components, biotic resistance, and abiotic tolerance were selected for descent. All spikes in each generation were bagged. Finally, a total of 197 lines of the F_7_ generation were produced from the pentaploid-derived population, and the 4D(4B) disomic substitution line YL-443 was selected from the F_7_ generation using mc-FISH. Figure 1 shows the procedure followed to develop YL-443.

Lanmai and CS were obtained from the Sichuan Academy of Agricultural Sciences (SAAS), China. AT23 was provided by Dr. A. Mujeeb-Kazi, International Maize and Wheat Improvement Center (CIMMYT), Mexico, in 1995 and kept in SAAS. The accession number in CIMMYT is AE. SQUARROSA (309).

### Mc-FISH Characterization

Multicolor fluorescence *in situ* hybridization was used to identify the chromosome constitutions of the introgression lines. Slides of root tips of Lanmai/AT23//Lanmai F_7_ generation and their parents Lanmai and AT23 were prepared as described by Zhang et al. (2018) and Kato et al. (2004). Two oligonucleotide probes, *Oligo-pSc119.2* (hybridization with B genome chromosomes) and *Oligo-pTa535* (hybridization with A and D genome chromosomes) (Tang et al., 2014), were labeled with 6-carboxyfluorescein (6-FAM) or 6-carboxytetramethylrhodamine (TAMRA) at the 5′-end (synthesized by Invitrogen Biotechnology Co. Ltd., Shanghai, China) and hybridized to the slides as reported by Fu et al. (2015). After hybridization, the slides were stained and imaged following the procedures by Zhang et al. (2020).

### Yield Component Traits Survey

The yield component traits of Lanmai, YL-724, and YL-443 was examined at the Pidu experiment station of SAAS, Sichuan, China, in 2020 and 2021. A randomized complete block design was arranged with three replications. The seeds were sown as described by Wan et al. (2015). After maturity, the plant height (PH), spike length (SL), spikelet number per spike (SNS), floret number per spike (FNS), kernel number per spike (KNS), and seed setting rate (SR) (calculated through dividing KNS by FNS) were recorded with 10 replications of each material.

Mean ± SD was calculated using IBM SPSS Statistics (Version 26) (George and Mallery, 2019). Two-way ANOVA followed by Bonferroni's multiple comparisons test was performed using GraphPad Prism (Version 8.0.1) (Mavrevski et al., 2018) to compare the variances among different years and lines for all traits.

### Morphological Trait Analysis

Hairy leaf sheath and auricles are the morphological trait studied in this study [Note: Most *Ae. tauschii* accessions possess this trait which could be transferred into common wheat through SHW-involved crossing (Yang et al., 1999), and its associated QTL has been mapped on 4DL (Dobrovolskaya et al., 2007; Wan et al., 2015). Therefore, hairy leaf sheath and auricles mark the authenticity of 4D(4B) substitution line]. During phenotyping, pubescence was examined on the leaf sheath and auricles of lines Lanmai, AT23, YL-443, and YL-724.

### Adult Plant Resistance Assessment of Stripe Rust

Stripe rust was caused by *Puccinia striiformis* f. sp. *tritici* (Pst). Its resistance genes *YrAS2388* and *Yr28*, which are believed to be the same gene, have been mapped on chromosome 4D in *Ae. tauschii* (Singh et al., 2000; Huang et al., 2011; Liu et al., 2013; Zhang R. et al., 2019). Combining with various stripe rust resistance phenotypes screened in the field, the PCR primer of *YrAS2388* that was specified in Zhang C. et al. (2019) was used to identify the Pst resistance gene. The PCR and thermal cycles were performed as described by Zhang C. et al. (2019).

Adult plant resistance (APR) tests of stripe rust were conducted via a field trial at the Pidu experiment station of SAAS, Sichuan, China, in 2021. Each field plot of lines Lanmai, AT23, YL-443, and YL-724 and spreader rows were sown as reported by Rouse et al. (2011). The mixed urediniospores of Pst races, CYR32, CYR33, and CYR34 (Wan et al., 2007; Chen et al., 2009; Yang et al., 2019) that are currently prevalent in China, were inoculated onto the highly susceptible wheat Chuanyu12 (Chengdu Institute of Biology of Chinese Academy of Sciences, Sichuan, China), which was used as a spreader and control. Adult plant infection types (IT) were recorded when rust development reached 90% on Chuanyu12 using a 0–4 scale as described by Chen et al. (2009) and divided into five categories: immune (I, 0), near immune (NI, 0), high resistance (HR, 1), moderate resistance (MR, 2), moderate susceptibility (MS, 3), and susceptible (S, 4). The IT was scored and recorded once every 10 days, making a total of three times of recording for all field plots. The Pst races were provided by the Institute of Plant Protection, SAAS and the Institute of Plant Protection, Gansu Academy of Agricultural Sciences.

### KASP Markers Screening of 4D and 4B Chromosomes

The Kompetitive Allele-Specific PCR (KASP) assay (Grewal et al., 2020) was used to screen the 4D and 4B chromosomes in this study. Each SNP-specific KASP primer set contains two allele-specific competing forward primers with unique tail sequences and one reverse primer. A total of 15 primer sets for chromosomes 4B and 16 primer sets for 4D were chosen based on the SNPs discovered on 4 hexaploid wheat and 23 wild relatives (Grewal et al., 2020) (Invitrogen Biotechnology Co. Ltd., Shanghai, China), to screen the chromosome substitution line YL-443. The parental lines Lanmai and AT23 and the landrace CS were used as controls. The KASP reaction system of 2 μl was modified from the “KASP genotyping chemistry User guide and manual” (https://biosearch-cdn.azureedge.net/assetsv6/KASP-genotyping-chemistry-User-guide.pdf), containing 1 μl of 2× KASP Master mix (standard ROX) (LGC Biosearch Technologies, Shanghai, China), 0.028 μl of primer mix (10 pmol/μl of each primer), and 1 μl of DNA (50 ng/μl). The KASP thermal cycling program was set based on the instructions in the user guide and manual.

All reactions were conducted using the GeneMatrix system (HC Scientific LLC., Chengdu, China). The collected data were analyzed by the companion Matrix Master Data Analysis & Management System.

## Results

### Chromosomal Characterization

**Figure 2:**
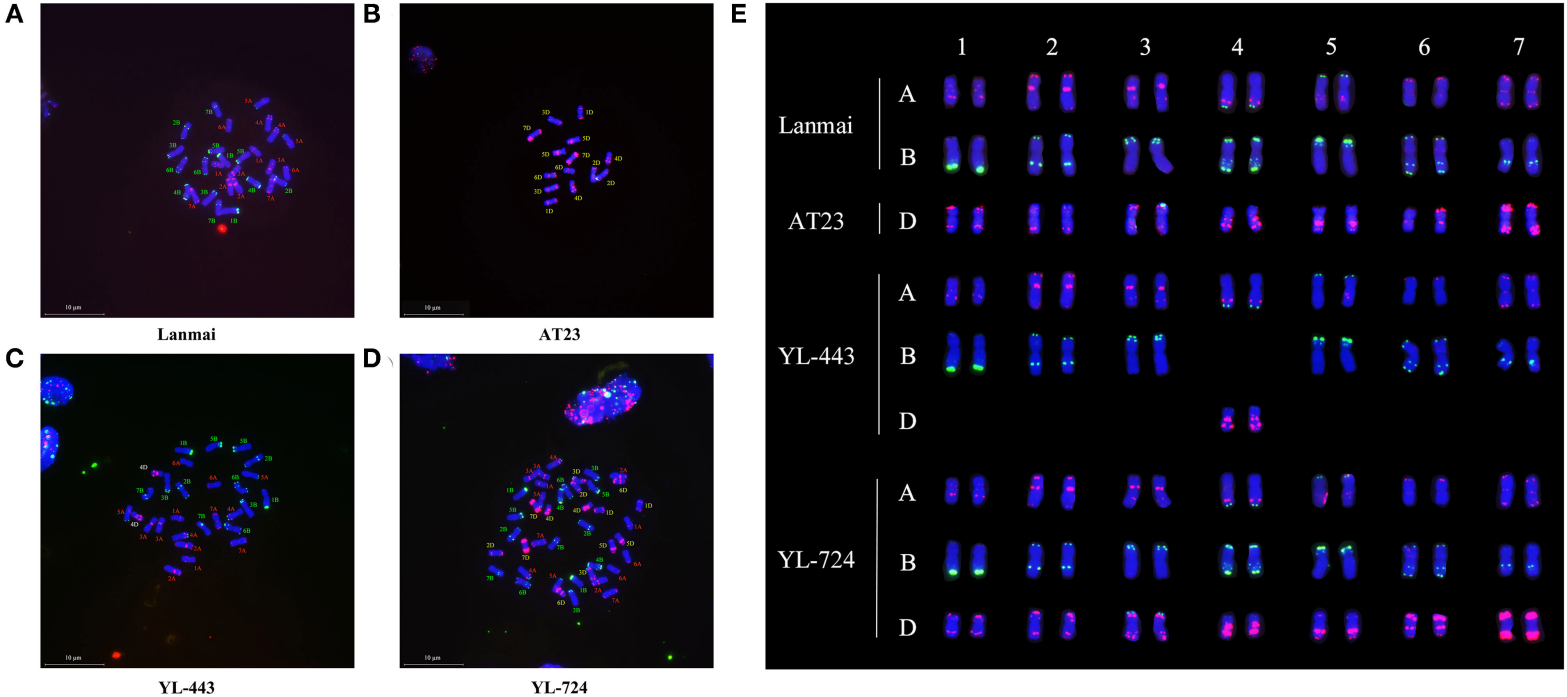
The mc-FISH karyotypes of Lanmai, AT23, YL-443, and YL-724 hybridized with probes *Oligo-pSc119.2* (green) and *Oligo-pTa535* (red). **(A)** Durum wheat Lanmai; **(B)** *Ae. tauschii* AT23; **(C)** Lanmai 4D(4B) disomic substitution line YL-443; **(D)** Lanmai/AT23 amphiploid line YL-724; **(E)** Comparison of chromosome constitution of all lines through cutting out chromosomes.

The results of mc-FISH detection of the mitotic spread chromosomes of Lanmai, AT23, YL-443, and YL-724 via probes *Oligo-pSc119.2* and *Oligo-pTa535* are shown in Figure 2. The FISH karyotype of the SHW line YL-724 showed 42 chromosomes (Figures 2D,E), demonstrating an accurate distinction of the durum wheat and *Ae. tauschii* chromosomes using the above probes. The *Oligo-pTa535* (red) highlights a pair of 4D chromosomes of *Ae. tauschii* with strong hybridization signals near the telomeric region of the chromosomal long arm and the pericentromeric region (Figures 2B,C,E). On the same chromosome, it also has an *Oligo-pSc119.2* (green) signal at the end of the short arm (Figures 2B,C,E). Compared with the karyotype of CS (Tang et al., 2014), Lanmai (Figures 2A,E), AT23 (Figures 2B,E), and YL-724 (Figures 2D,E), line YL-443 (Figures 2C,E) clearly showed as a 4D(4B) disomic substitution line that contained a pair of 4D chromosomes plus the other 26 durum wheat chromosomes.

### Yield Component Traits Survey

**Figure 3:**
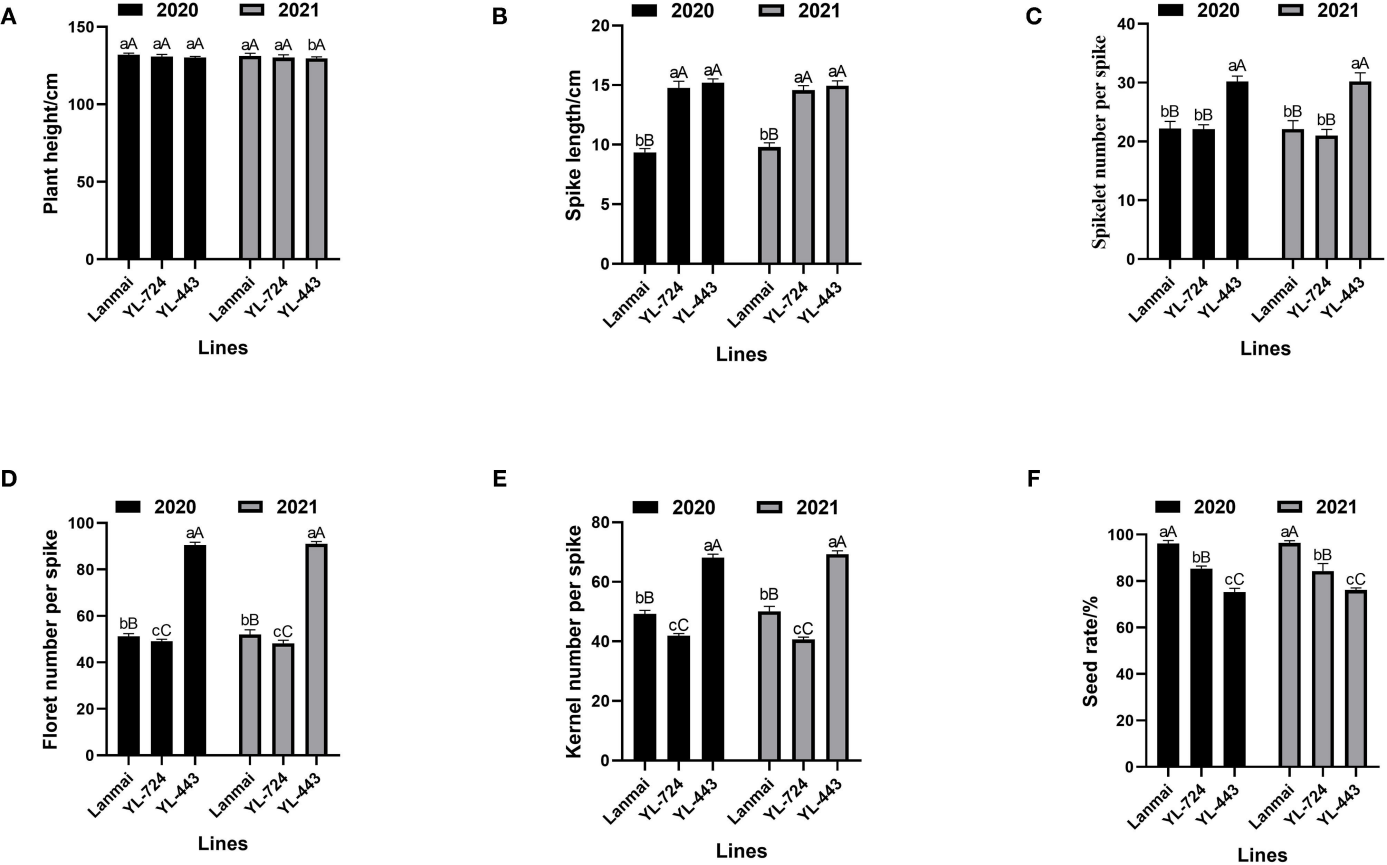
ANOVA of yield component trait of durum wheat Lanmai, Lanmai/AT23 amphiploid line YL-724, *Ae. tauschii* AT23, and Lanmai 4D(4B) disomic substitution line YL-443. **(A)** Plant height comparison. **(B)** Spike length comparison. **(C)** Spikelet number comparison. **(D)** Comparison of floret number per spike. **(E)** Comparison of kernel number per spike. **(F)** Seed setting rate comparison. The same lowercase letters indicate that the difference among lines was not significant. The same uppercase letters mean the difference between years was not significant.

**Figure 4:**
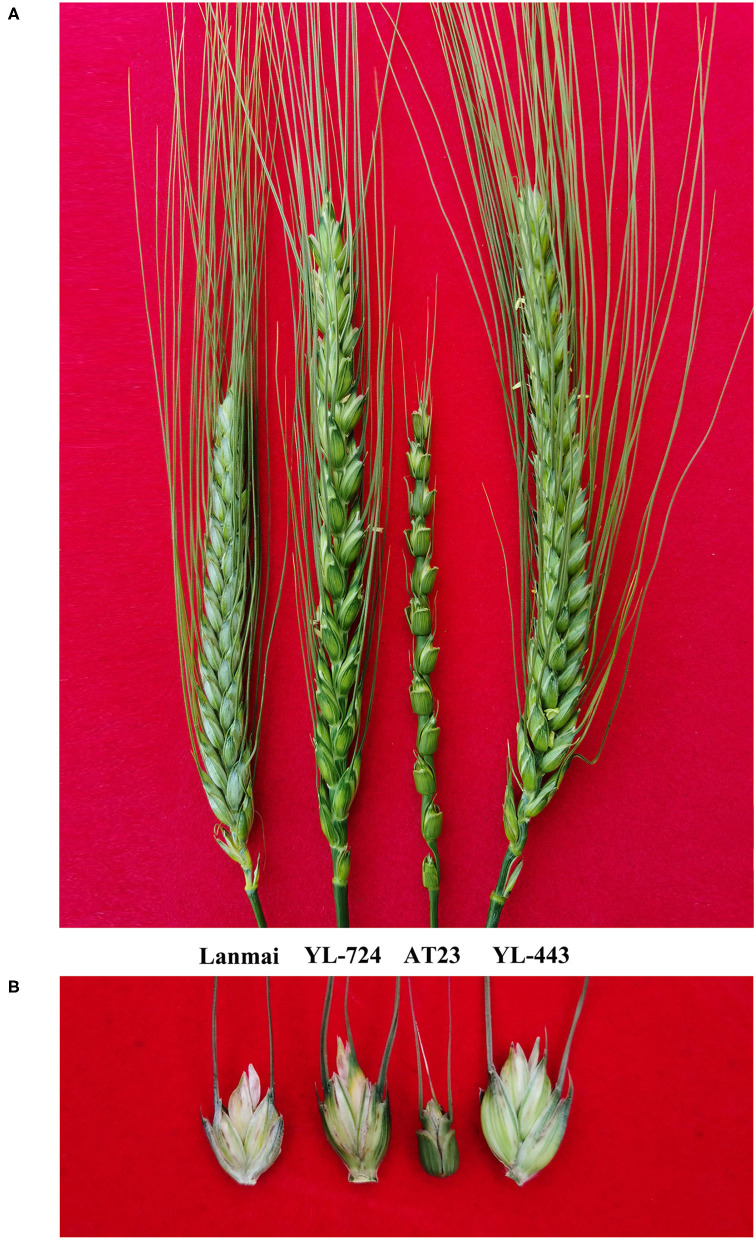
The spike and spikelet comparison of durum wheat Lanmai, Lanmai/AT23 amphiploid line YL-724, *Ae. tauschii* AT23, and Lanmai 4D(4B) disomic substitution line YL-443. **(A)** Spike comparison. **(B)** Spikelet comparison.

The yield component traits of Lanmai, YL-724, and YL-443 were phenotyped in 2020 and 2021 (Table 1). For all lines, there was no significant difference between years based upon the ANOVA results (Figure 3). All lines almost had the same plant height (Figure 3A). Even though the lines had similar spike length YL-443 displayed more spikelets per spike than that of YL-724 (Figures 3B, 4A,B). Meanwhile, YL-443 had a significantly higher spikelet number per spike (SNS) and floret number per spike (FNS) than the other lines (Table 1; Figures 3C,D, 4A,B). Compared with Lanmai, YL-443 had SNS and FNS higher than Lanmai by 36.3% and 75.9%, respectively. Although YL-443 had a lower seed setting rate than that of Lanmai and YL-724, it still had the highest kernel number per spike among all lines (Table 1; Figures 3E,F).

**Table 1:** Summary of yield component trait.

**Years**	**Lines**	**PH/cm**	**SL/cm**	**SNS**	**FNS**	**KNS**	**SR/%**
2020	Lanmai	132.1 ± 1.0aA	9.4 ± 0.3bB	22.2 ± 1.2bB	51.2 ± 1.1bB	49.2 ± 1.2bB	96.1 ± 1.3aA
	YL-724	130.8 ± 1.3aA	14.8 ± 0.5aA	22.1 ± 0.7bB	49.1 ± 0.8cC	41.9 ± 0.7cC	85.3 ± 1.1bB
	YL-443	130.2 ± 0.7aA	15.2 ± 0.3aA	30.2 ± 0.9aA	90.5 ± 1.1aA	68.1 ± 1.1aA	75.3 ± 1.5cC
2021	Lanmai	131.4 ± 1.5aA	9.8 ± 0.3bB	22.1 ± 1.4bB	52.0 ± 1.9bB	50.1 ± 1.6bB	96.4 ± 1.0aA
	YL-724	130.2 ± 1.6aA	14.6 ± 0.4aA	21.0 ± 1.0bB	48.2 ± 1.2cC	40.6 ± 0.8cC	84.3 ± 3.1bB
	YL-443	129.7 ± 1.0bA	15.0 ± 0.4aA	30.2 ± 1.4aA	91.0 ± 1.0aA	69.3 ± 1.1aA	76.2 ± 0.8cC
LDN^*^	129				34.8		
4D(4B) substitution line^*^	88				3.9		

* *Data reported by Joppa (1987) and Joppa and Williams (1988)*.

*PH, plant height; SL, spike length; SNS, spikelet number per spike; FNS, floret number per spike; KNS, kernel number per spike; SR, seed setting rate*.

*The same lowercase letters indicate that the difference among lines was not significant. The same uppercase letters mean that the difference between years was not significant*.

### Morphological Trait Analysis

**Figure 5:**
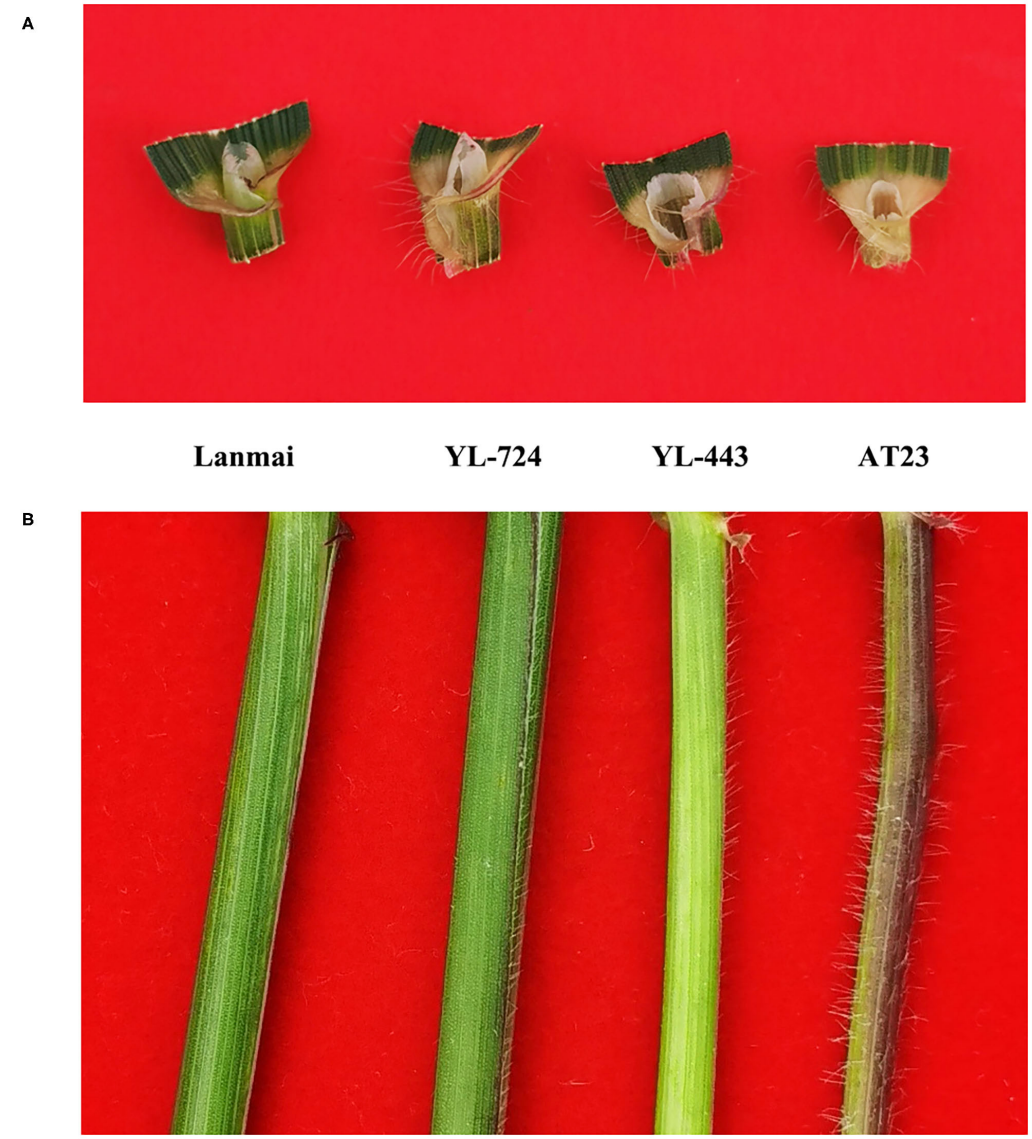
Comparison of leaf sheath and auricles hairiness among durum wheat Lanmai, Lanmai/AT23 amphiploid line YL-724, 4D(4B) disomic substitution line YL-443, and *Ae. tauschii* AT23. **(A)** Hairy auricles comparison. **(B)** Hairy leaf sheath comparison.

Since our previous work has mapped the QTLs for hairy leaf sheath and auricles on chromosome 4DL (Wan et al., 2015), indicating that the 4D(4B) substitution line YL-443 should contain the hairy leaf sheath and auricles characteristics of AT23. The comparison of leaf sheath and auricles hairiness among lines Lanmai, AT23, YL-724, and YL-443 is shown in Figure 5. Lanmai had a glabrous leaf sheath and auricles, while AT23, YL-724, and YL-443 were all pubescent. This special characterization of YL-443 verified its authenticity as a 4D(4B) chromosome substitution. It is worth noting that the hairy leaf sheath and auricles characteristics can be used for assisting selection in crosses involving YL-443.

### Stripe Rust Resistance Evaluation

**Figure 6:**
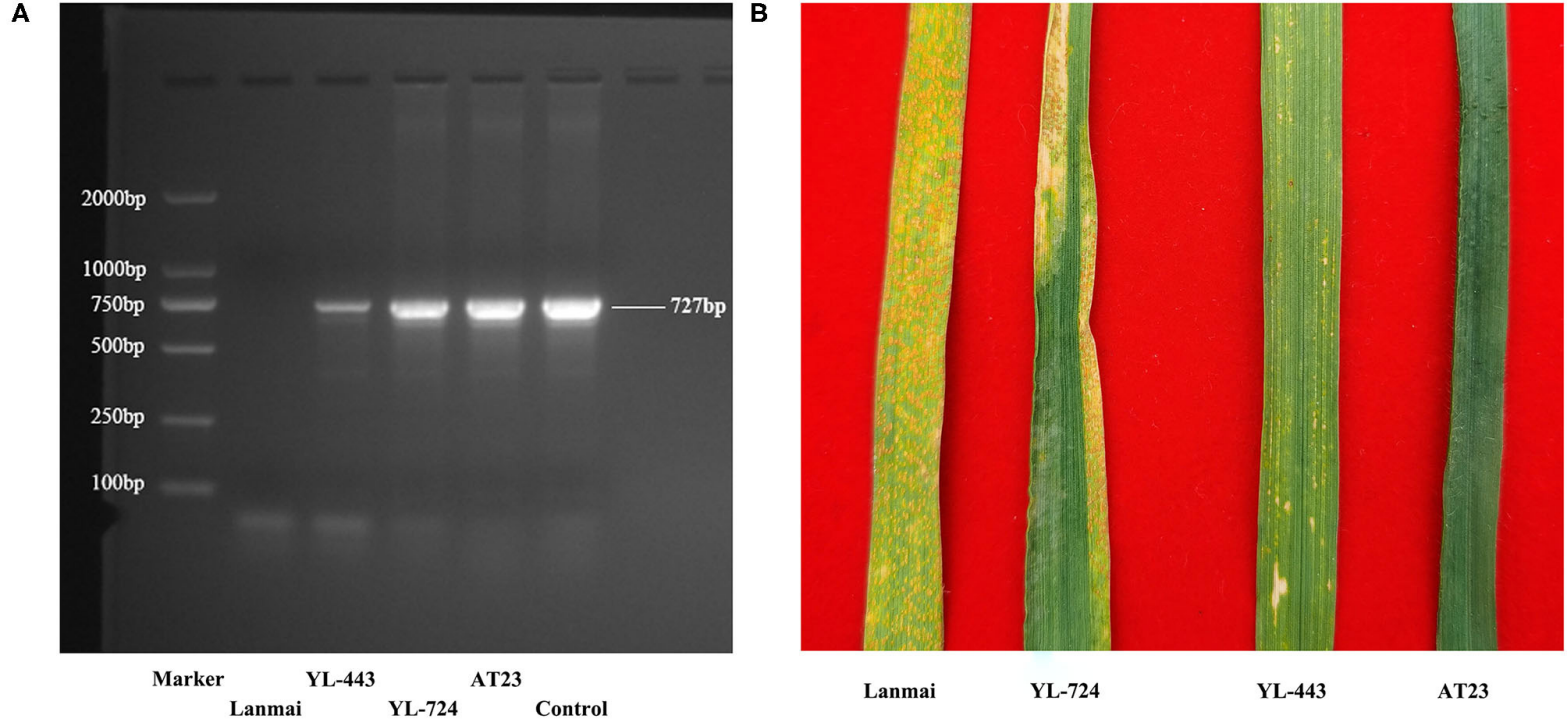
The Yr28 gene amplification results and different APR phenotypes of durum wheat Lanmai, Lanmai/AT23 amphiploid line YL-724, 4D(4B) disomic substitution line YL-443, and *Ae. tauschii* AT23 for stripe rust. **(A)** The Yr28 gene amplification results with positve products (727 bp) in all plants except Lanmai. **(B)** The APR test results of Lanmai (S), YL-724 (MR), YL-443 (HR), and AT23 (I) for stripe rust resistance. I, immune; NI, near immune; HR, high resistance; MR, moderate resistance; MS, moderate susceptibility; S, susceptible.

The stripe rust resistance gene *Yr28* has been mapped on chromosome 4D in *Ae. tauschii* (Singh et al., 2000; Huang et al., 2011; Liu et al., 2013; Zhang R. et al., 2019). As shown in Figure 6A, PCR using DNA from lines YL-443, YL-724, and AT23 produced a specific amplicon (727 bp) of *Yr28*, while PCR using DNA from Lanmai did not. This result indicated that the *Yr28* gene has been transferred from *Ae. tauschii* AT23 to YL-724 and then to YL-443.

To identify the infection type of YL-443, inoculation of stripe rust was conducted in the field using the currently prevalent Pst races in China including CYR32, CYR33, and CYR34. *Ae. tauschii* AT23 showed an immune response to all Pst races, whereas durum wheat Lanmai was susceptible (Figure 6B). The amphiploid line YL-724 displayed an MR phenotype (Figure 6B), which is similar to another SHW line reported by Zhang R. et al. (2019). The developed substitution line YL-443 showed high resistance to all Pst races (Figure 6B). This indicates that the *Yr28* gene can fully express in the 4D(4B) disomic substitution line in a tetraploid background but is partially suppressed in a hexaploid genetic background.

### KASP Markers Identification

Results of the identification of the chromosomal substitution using the released specific KASP markers of chromosomes 4D (15 sets) and 4B (16 sets) developed by Grewal et al. (2020) were shown in Supplementary Data S1. For the 15 4B-specific markers, YL-443 and AT23 failed to give a positive result, while YL-724 showed the same results as plant Lanmai except WRC500 (with no result for Lanmai). Among the 16 4D-specific markers, seven showed no result for all plants, possibly due to the genetic variations compared with marker-based reference germplasm. For the remaining nine markers, Lanmai (without chromosome 4D) showed no positive results, while YL-443 and YL-724 both showed the same genotype as their parent AT23 and one marker WRC531 showing no result with CS. The results meant that the 4D(4B) substitution was verified by 23 specific KASP markers including 9 markers for 4D and 14 markers for 4B, suggesting that these markers can be used for 4D introgression line selection during durum wheat breeding.

## Discussion

The D genome addition and inter/intra-specific introgressions cause a substantial difference between durum and bread wheat at the whole genome level (Dubcovsky and Dvorak, 2007; Cheng et al., 2019). Consequently, many end-use-related trait variations can be inserted, such as kernel hardness, grain protein content (gliadins and glutenins), and yellow pigment content (Mastrangelo and Cattivelli, 2021). On the other hand, the D-genome contains a high number of biotic resistance and abiotic tolerance genes, while the A or B genome usually lacks these genes (Padmanaban et al., 2017). Therefore, strategies to incorporate new alleles or traits from the D genome into the A or B genome have been followed for improving durum wheat. Previous researches have mainly focused on introgression by *ph1b-* and *ph1c*-mediated homologous recombination for developing translocations with chromosome fragments of the D genome (Sears, 1977; Ceoloni et al., 1995, 1996; Vitellozzi et al., 1997; Gennaro et al., 2012; Boehm et al., 2017; Morris et al., 2019; Sissons et al., 2019) or by crossing CS nullitetras with the durum wheat Langdon for developing D-genome substitutions (Joppa et al., 1983; Joppa and Williams, 1988). Most of the D-chromosomes introduced into durum wheat come from common wheat, such as CS (Joppa and Williams, 1988; Morris et al., 2011; Han et al., 2014, 2016). Chromosomes 4T-b and 4H^ch^ from *Am. muticum* or *H. chilense* were also transferred into durum wheat, resulting in the development of 4T-b(4B) and 4H^ch^(4B) disomic substitution lines, respectively (Calderón et al., 2012; Othmeni et al., 2019).

Here, we reported a new procedure for developing introgression and/or substitution lines by using an allohexaploid wheat crossed with its tetraploid donor wheat. These introgressed segments or chromosomes under a single genetic background have an advantage for mapping genes and mining gene functions. By using this new procedure, a novel durum 4D(4B) disomic substitution line possessing a pair of 4D chromosomes from *Ae. tauschii* was developed.

According to the data reported by Joppa (1987) and Joppa and Williams (1988), durum wheat LDN had similar plant height and kernel number per spike, and its 4D(4B) substitution line with chromosome 4D from CS showed significantly lower plant height and kernel number per spike (Table 1). In contrast, due to the 4D chromosome compensation effect, the developed substitution line in this study has a super-large spike with a significantly (*P* < 0.001) increased spikelet number per spike and floret number per spike outperforming the Lanmai by 36.3 and 75.9%, respectively (Table 1; Figures 3C,D). These results indicated that the chromosome 4D of AT23 has a better compensatory effect than that of CS in a tetraploid background and possesses new genes for supernumerary spikelets. Although YL-443 had a lower seed setting rate than that of Lanmai and YL-724, its kernel number per spike was still the highest among all lines (Table 1; Figures 3E,F). These results demonstrate that the substitution line YL-443 can be further used for high-yield breeding by improving the seed setting rate.

To date, a range of genes or genetic loci that are responsible for supernumerary spikelets have been mapped on different chromosomes, such as 2A, 2B, 2D, 5B, 6A, 6B, 7A, and 7B (Li et al., 2012; Dobrovolskaya et al., 2015; Echeverry-Solarte et al., 2015; Kuzay et al., 2019; Ma et al., 2019; Rizwan et al., 2020; Du et al., 2021), but none has been reported on chromosome 4D. This study is the first report that chromosome 4D has strong effects on supernumerary spikelets. Further work is required to identify the gene underlying the supernumerary spikelets trait of the 4D chromosome of YL-443. In parallel, our previous research mapped a major hairy leaf sheath QTL on 4DL that were originated from *Ae. tauschii* and related with the enhanced grain yield trait in a SHW-derived wheat variety CM42 (Wan et al., 2015; Li et al., 2018). This indicates that the hairy leaf sheath genes in YL-443 may be related to its enhanced yield component traits, thus are potentially valuable in breeding aiming for yield improvement.

The resistance evaluation of stripe rust showed that the amphiploid line YL-724 had lower resistance than that of the developed substitution line YL-443, indicating a suppressed response to Pst races under a hexaploid genetic background, which was consistent with the results reported by Zhang C. et al. (2019). The stripe rust resistance gene *Yr28* from the 4D chromosome of *Ae. tauschii* AT23 was transferred and completely expressed in the developed substitution line YL-443. Previous research also mapped the major salinity-tolerant gene *Kna1* (Lindsay et al., 2004) and Al-tolerant gene *TaALMT1* (Han et al., 2014, 2016) on 4D, which means that the 4D chromosome introduction may enhance the abiotic stress tolerance as well. Therefore, the 4D(4B) disomic substitution line YL-443 can potentially be an elite germplasm for enhancing the Pst resistance and Al tolerance of durum wheat. Moreover, YL-443 could also be used for cross-hybridization with rye for generating primary hexaploid triticale lines containing part of D genomes (Kwiatek and Nawracała, 2018). The *Yr28*-specific PCR marker, 4D chromosome-specific KASP markers, and special pubescent leaf sheath and auricles features can be utilized for assisting selection.

Considering the substantial and positive variations of the developed Lanmai-AT23 4D(4B) substitution line in this study, some tetraploid recombinant inbred lines (RIL) are currently under development by using YL-443 to cross with the CS and a synthesized hexaploid wheat Lanmai/AT18 (*Ae. tauschii* var. *typica* accession AT18). These RIL lines will help us understand the loss of 4D-chromosome functional genes after allohexaploid hybridization through fine-mapping the 4D-chromosome genes carried by *Ae. tauschii* AT23, such as genes that control supernumerary spikelets. On the other hand, although the whole chromosome substitution line reported in this study showed a range of enhanced phenotypes, it does not mean that whole chromosome substitution can be expanded to all 14 chromosomes for durum wheat trait enhancement. There are insufficient studies to support such a proposal. A thorough characterization is needed to judge the value of any substitution line in breeding.

## Conclusion

A durum wheat-*Ae. tauschii* 4D(4B) disomic substitution line YL-443 carrying the 4D chromosome with high stripe rust resistance, large spike, and unique pubescent leaf sheath and auricles features was developed and verified by mc-FISH, *Yr28* gene-specific PCR, and 4D- and 4B-specific KASP assays. The developed line can potentially be used for future durum wheat yield improvement. The *Yr28*-specific PCR marker, 4D chromosome-specific KASP markers, and pubescent leaf sheath and auricles phenotype can be used to assist selection in breeding.

## Data Availability Statement

The datasets presented in this study can be found in online repositories. The names of the repository/repositories and accession number(s) can be found in the article/Supplementary Material.

## Author Contributions

WY, WM, and YZ conceptualized the study. FY, QW, NY, SY, YW, and JZ contributed to the methodology. FY, QL, NY, and HL contributed to the formal analysis. NY, ZL, and WY contributed to the investigation. FY, QW, and SY contributed to the data curation. HW and WY contributed to the resources. XF, WY, and YZ contributed to the funding acquisition. JL and HW contributed to the project administration. FY contributed to the manuscript writing (original draft). WM, XF, and WY contributed to the manuscript writing (reviewing and editing). All authors contributed to the article and approved the submitted version.

## Funding

This work was funded by the China Scholarship Council, the Accurate Identification Project of Crop Germplasm from Sichuan Provincial Finance Department, the National Natural Science Foundation of China (Nos. 31870360, 31470305), the Second Tibetan Plateau Scientific Expedition and Research Program (STEP) (No. 2019QZKK0303), and the China Agriculture Research System of MOF and MARA (CARS-03). This work was supported by Sichuan Agricultural University in China, Sichuan Academy of Agricultural Sciences in China, and Murdoch University in Australia.

## Conflict of Interest

The authors declare that the research was conducted in the absence of any commercial or financial relationships that could be construed as a potential conflict of interest.

## Publisher's Note

All claims expressed in this article are solely those of the authors and do not necessarily represent those of their affiliated organizations, or those of the publisher, the editors and the reviewers. Any product that may be evaluated in this article, or claim that may be made by its manufacturer, is not guaranteed or endorsed by the publisher.
